# Mucin Dynamics in Intestinal Bacterial Infection

**DOI:** 10.1371/journal.pone.0003952

**Published:** 2008-12-17

**Authors:** Sara K. Lindén, Timothy H. J. Florin, Michael A. McGuckin

**Affiliations:** 1 Mucosal Diseases Program, Mater Medical Research Institute, Mater Health Services, South Brisbane, Queensland, Australia; 2 Mucosal Immunobiology and Vaccine Center, Sahlgrenska Academy, Gothenburg University, Gothenburg, Sweden; 3 Department of Medicine, University of Queensland, Brisbane, Queensland, Australia; University of Cambridge, United Kingdom

## Abstract

**Background:**

Bacterial gastroenteritis causes morbidity and mortality in humans worldwide. Murine *Citrobacter rodentium* infection is a model for gastroenteritis caused by the human pathogens enteropathogenic *Escherichia coli* and enterohaemorrhagic *E. coli*. Mucin glycoproteins are the main component of the first barrier that bacteria encounter in the intestinal tract.

**Methodology/Principal Findings:**

Using Immunohistochemistry, we investigated intestinal expression of mucins (Alcian blue/PAS, Muc1, Muc2, Muc4, Muc5AC, Muc13 and Muc3/17) in healthy and *C. rodentium* infected mice. The majority of the *C. rodentium* infected mice developed systemic infection and colitis in the mid and distal colon by day 12. *C. rodentium* bound to the major secreted mucin, Muc2, *in vitro*, and high numbers of bacteria were found in secreted MUC2 in infected animals *in vivo*, indicating that mucins may limit bacterial access to the epithelial surface. In the small intestine, caecum and proximal colon, the mucin expression was similar in infected and non-infected animals. In the distal colonic epithelium, all secreted and cell surface mucins decreased with the exception of the Muc1 cell surface mucin which increased after infection (p<0.05). Similarly, during human infection *Salmonella St Paul*, *Campylobacter jejuni* and *Clostridium difficile* induced MUC1 in the colon.

**Conclusion:**

Major changes in both the cell-surface and secreted mucins occur in response to intestinal infection.

## Introduction

Bacteria such as enteropathogenic *Escherichia coli* (EPEC), enterohaemorrhagic *E. coli* (EHEC), *Salmonella enteritidis* and *Clostridium difficile* are major causes of infectious diarrhea in humans worldwide. EPEC and EHEC develop a commensal, rather than pathogenic, interaction within the mouse host [1], [2]. *C. rodentium* is a natural mouse pathogen that is related to, and uses the same molecular mechanisms of type III secretion and attaching and effacing lesions as human EPEC and EHEC, to colonise the epithelial cells of the gut, hence providing a good *in vivo* model for gastroenteritis due to these bacteria [3], [4].

The gastrointestinal tract is lined by a continuously secreted mucus layer formed by high molecular mass oligomeric mucin glycoproteins. This mucus layer moves to clear trapped material. In the healthy human intestine, MUC2 is the main secreted mucin making up the mucus layer, whereas in the stomach MUC5AC and MUC6 are produced. Under the mucus layer, the cell-surface mucins are a dominant feature of the apical surface of all mucosal epithelial cells. Cell-surface mucins are likely to play an important role in mucosal defense since they may provide both a barrier and reporting function, and we have demonstrated increased pathology following gastrointestinal infection in mice lacking the Muc1 cell surface mucin [5], [6]. These filamentous molecules extend further than most other cell surface structures [7]. The intestine produces the MUC1, MUC3, MUC4 MUC12, MUC13 and MUC17 cell surface mucins [8], [9].

Microbial products and inflammatory cytokines stimulate increased production of mucins by mucosal epithelial cells, which can effect a massive discharge of mucin in response to stimuli [10], [11], [12], [13], [14], [15], [16], [17], [18], [19], [20], [21]. Stimulated mucin release occurs rapidly and is accompanied by a hundredfold expansion of the secretory granules upon hydration. Several pathogens have been shown to interact with mucins [22], [23], [24], [25], [26], including EPEC and EHEC which bind to bovine mucins [27]. Upregulation of MUC3 expression in colonic cells has been correlated with decreased binding of EPEC [28], [29]. The ability of human milk to limit gastrointestinal bacterial and viral infections has been attributed in part to the presence of large amounts of cell surface mucins, chiefly MUC1 and MUC15 [30], [31], [32]. Consistent with an important role for Muc1 upregulation in the intestine limiting infection, Muc1^−/−^ mice have a higher rate of systemic infection in a murine *Campylobacter jejuni* model of gastroenteritis [6]. Many pathogens have evolved mechanisms to subvert the mucin barrier, for example the StcE zinc metalloprotease secreted by EHEC is a mucinase [33].

Goblet cell depletion in infection has been reported previously [34], however, no comprehensive study of expression of all mucins in an animal infection model has been performed. We map the localization along the intestinal tract of all murine mucins for which antibodies are available: Muc1, Muc2, Muc4, Muc5AC, Muc13 and Muc3 (orthologue of human MUC17, therefore hereafter referred to as Muc17. There is currently no Muc3 orthologue annotated in the mouse genome. However, a peptide identical to a peptide from the human MUC3 mucin has been identified using proteomics on mouse mucins, suggesting the presence of the mouse orthologue to this mucin [35]). In a murine *C. rodentium* infection model, we demonstrate substantial changes in the amount of virtually all intestinal mucins after 12 days of infection. We further showed mucin binding to *C. rodentium*, demonstrating that the release of mucins has the capacity to remove bacteria from the epithelial surface.

## Materials and Methods

### Animals

8–16 weeks old male129/SvJxC57BL/6 mice were housed under clean conventional conditions, and allowed free access to sterilized food and water. All experiments were approved by the University of Queensland Animal Experimentation Ethics Committee (approval no 059/07). Colonies routinely tested negative for murine viral and bacterial pathogens.

### Infection of mice

*Citrobactum rodentium* strain ICC169 was grown on Lauria-Bertani agar for 20 h at 37°C. Bacteria harvested from plate cultures were suspended in warmed Lauria-Bertani broth. 8 mice were orally inoculated with 10^7^ colony forming units (CFU) and sacrificed after 12 days by cervical dislocation. Duplicate samples of duodenum, caecum, small intestine and proximal, mid and distal colon were dissected and collected in either broth or 10% formalin. To assess the number of CFU/g tissue, tissue and fecal samples (collected as defecated samples) were homogenized in broth, serially diluted, plated onto McConkey's selective agar, and grown for 20 h at 37°C. CFU were enumerated by counting *C. rodentium* ∼1 mm diameter fuschia-coloured colonies. The mice were weighed and diarrhea was scored every second day.

### Histological assessment

For analysis of colitis, formalin fixed tissue sections of the small intestine, caecum, proximal and distal colon stained with hematoxylin/eosin were coded to blind the analysis, and the entire section was systematically scored: aberrant crypt architecture (0–5), increased crypt length (0–3), goblet cell depletion (0–3), general leukocyte infiltration (0–3), lamina propria neutrophil counts (0–3), crypt abscesses (0–3), and epithelial damage and ulceration (0–3) were scored (full details in Table S1).

### Human biopsies

Individuals with acute diarrhoea are not usually subjected to colonoscopic examination and biopsy, but archival biopsy material was available from seven patients with acute gastroenteritis. These patients included four previously healthy individuals and three patients with underlying inflammatory bowel disease, as described in Table 1. For controls, biopsies were examined from the same IBD patients when there was no detectable bacterial infection or from the same distal colonic site from age and sex matched healthy individuals (Table 1). The archived biopsies were collected and treated in accordance with the Australian National Statement on Ethical Conduct in Human Research (2007) (http://www.nhmrc.gov.au/publications/synopses/e72syn.htm) guidelines for archived pathological material. There is no consent process required for this type of use. The biopsies were collected between 1/06 and 1/08 for diagnostic histopathology as requested by the attending doctor. The samples were processed by the Mater Health Services Pathology Service as requested by the attending doctor. The paraffin blocks are kept on level 6 of the Mater Adult Hospital, which is where the Mater Health Services Pathology Service is housed.

**Table 1:** Human biopsies used for investigating MUC1 induction in infectious colitis.

Infection patient			Control patient	Infectious agent
Age	Sex	IBD status*		
59	F	No	Different patient: matched for age, sex and biopsy site	*S.* St Paul
71	F			*C. jejuni*
23	F			*C. jejuni*
46	M			*C. jejuni*
19	M	CD	Same patient and biopsy site>12 months from acute infection	*C. difficile*
47	F	CD		*C. difficile*
27	M	UC		*S. enteritidis*

* CD = Crohn's disease, UC = ulcerative colitis.

### Antibodies

The MUC1 antibody (CT2[36]) and the Muc17/Muc3-S2 antibody (raised against the peptide KYTPGFENTLDTVVKNLETKIKNAT [35] were kind gifts from Prof. S. Gendler, Scottsdale, USA, and Prof. G. Hansson, Gothenburg, Sweden. The polyclonal anti sera against the C-terminal sequence of MUC4 (hHA1-B-1) was a kind gift from Prof. K. Carraway, Miami, USA. The MUC5AC antibody (45MI) was purchased from Sigma [37]. Polyclonal antisera (rM13.C and MM2-2) against the cytoplasmic tail of the human MUC13 and murine Muc2 was raised in rabbit using bovine serum albumin conjugated peptides CMQNPYSRHSSMPRPDY and CPEDRPIYDEDLKK, respectively [38], [39], and then purified on a HiTrap NHS-activated HP column (Amersham Biosciences, Uppsala, Sweden) coupled to the same peptides according to manufacturers instructions. The monospecificity of the CT2 and rM13.C antibodies were demonstrated by that gastric and intestinal specimens from Muc1 and Muc13 knockout mice were negative for staining with the respective antibodies. The staining pattern of the hHA1-B-1 antisera was similar to that previously described for MUC4 in human tissue [40], [41], and the pre-immune sera from the hHA1-B-1 rabbit did not stain any tissue. The 45M1 antibody recognizes MUC5AC (and no other molecules in gastric tissue extracts) in human and rhesus monkey as demonstrated by that only fractions from density gradient centrifugation that has a density appropriate for MUC5AC (1.4 g/ml) are recognized with this antibody [37], and the mouse gastric tissue used here as a positive control demonstrated a strong surface epithelial cell staining, similar to that of the human and rhesus monkey gastric tissue[37], whereas the intestinal tissue was negative. Similarly, the MM2-2 antibody recognize material from intestinal tissue extracts in fractions of appropriate density from density gradient centrifugation of MUC2/Muc2, show a distinct goblet cell staining pattern in the intestine of mice similar to that demonstrated in human [39], and did not stain the gastric negative control tissue. The specificity of the Muc3-S2 antibody has been confirmed using mass spectrometry [35].

### Immunohistochemistry

Mouse samples: Formalin-fixed, paraffin-embedded tissue sections (4 µm) of archived biopsies from mouse stomach, duodenum, caecum, small intestine and proximal-, mid- and distal colon were dewaxed and rehydrated. Antigen retrieval used for the detection of the different antibodies was either: 1) 10 mM citric acid, pH 6 at 95°C for 20 min (CT2, Muc17/Muc3-S2); 2) 10 mM citric acid, pH 6 at 95°C for 20 min followed by 10 mM 1,4-dithiothretiol in 0.1 M Tris/HCl buffer, pH 8 at 37°C for 30 min and then 25 mM iodoacetamine in the dark for 30 min (MM2-2); 3) High pH Antigen Retrieval Solution (Dako, RC20); or 4) Rodent decloaker (Biocare Medical, 45M1) at 80°C for 2 h. Sections were then treated with 3% (v/v) hydrogen peroxide for 10 min at room temperature. The sections were washed 3 times between all subsequent steps in phosphate buffered saline, pH 7.4 (PBS) containing 0.05% Tween-20. Non-specific binding was blocked by 10% Milk Diluent/Blocking solution (KPL, Maryland, USA) in PBS (CT2, MM2-2, Muc3-S2, hHAI-B-1 and RC20) or rodent block M (Biocare Medical, 45M1) for 30 min. The sections were incubated with a primary antibody diluted in 10% Milk Diluent/Blocking solution (KPL, Maryland, USA) in PBS containing 0.05% Tween-20 (CT2 1∶50, MM2-2 5 µg/ml, Muc17/Muc3-S2 1∶2000, hHAI-B-1 1∶200, 45M1 1∶1000 and rM13.C 30 µg/ml,) for 1 h, then incubated with anti-rabbit antibody conjugated to horseradish peroxidise (HRP), MM2-2, RC20) or anti-hamster antibody conjugated to biotin followed by streptavidin-HRP (CT2) for 1 h or with MM HRP-Polymer (Biocare Medical, 45M1) for 20 min. Bound antibody was visualized with diaminobenzidine for 10 min. The sections were counterstained with Harris's haematoxylin. Gastric sections were used as positive controls for CT2 and 45M1. Human samples: Formalin-fixed, paraffin-embedded tissue sections (4 µm) of archived biopsies were dewaxed and rehydrated. Sections were treated with 1% periodic acid to expose the epitope and then with 3% (v/v) hydrogen peroxide for 30 min at room temperature. The sections were washed 3 times between all subsequent steps in 0.15 M NaCl, 0.1 M Tris/HCl buffer (pH 7.4) containing 0.05% Tween-20. Non-specific binding was blocked by protein block (Dako) for 30 min. The sections were incubated with the BC2 antibody diluted in Antibody Diluent for 1 h, then incubated with Broad Spectrum Poly HRP Conjugate (Zymed Laboratories inc, San Fransisco, USA) for 10 min and then with diaminobenzidine for 10 min. The sections were counterstained with Harris's haematoxylin.

Staining was classified into one of four categories: high level of staining (approx. 90–100% of cells staining, score = 3), medium level of staining (25–90%, score = 2), low level of staining (1–25%, score = 1) and virtually no staining (<1%, score = 0). The scoring of the stain was performed by an individual blinded to the infection status of the mice. Two of the stains were also analyzed by a second individual blinded to the infection status of the animals, and the results between the two scorers were consistent.

### PAS/Alcian blue stain

De-waxed sections were immersed in 100% ethanol for 10 min, rinsed in water for 10 min, immersed in 3% acetic acid for 2 min and stained in 1% Alcian Blue 8GX in 3% acetic acid (pH 2.5) for 2.5 h. Non specific stain was removed with 3% acetic acid and rinsed in water for 10 min. The slides were then oxidized in 1% periodic acid in water at room temperature for 10 min, washed in water for 5 min, immersed in Schiff's reagent for 10 min, rinsed in water for 5 min and then three times in 0.5% sodium meta-bisulphite before a final wash in water. To reveal O-acetylated oligosaccharides sections were first treated with 0.1 M KOH for 30 min and then 1 mM periodic acid prior to the Schiff reagent.

### Antibody response against C. rodentium

*C. rodentium* were harvested into PBS, washed once and then sonicated 5 times while on ice with a 3 min interval between sonications. After sonication, the solution was centrifuged at 4000 g for 4 min. The supernatant was diluted to A280 = 0.4 and plated onto Maxisorb plates (Nunc). The microtiter plates were washed 3 times in PBS pH 7.4 containing 0.05% Tween-20 between all ensuing steps. Unbound sites were blocked with 1% bovine serum albumin (BSA) for 1 h. The microtiter plates were incubated with murine sera diluted 1∶100 in PBS containing 1% BSA and 0.05% Tween, pH 7.4, for 1 h at room temperature. The microtiter plates were incubated with HRP conjugated anti mouse Ig diluted 1∶2000. Bound secondary antibody was visualized using BD OptEIA TMB substrate reagent (BD, San Diego, USA). The reaction was stopped with 1 M sulphuric acid and absorbance at 450 nm was measured.

### C. rodentium binding to Muc2

The insoluble complex of Muc2 was extracted from murine intestinal mucosal scrapings as previously described [42]: mucosal scrapings were gently dispersed with a Dounce homogenizer in 6 M guanidinium chloride, 5 mM EDTA, 5 mM *N*-ethylmaleimide, 10 mM sodium phosphate buffer, pH 7.0, and left stirring at 4°C overnight. After centrifugation (21,000 g, 4°C, 60 min), the soluble material was removed, and the pellets were re-extracted twice, as described above. The final extraction residues (insoluble glycoprotein complex) were solubilized by reduction in 6 M guanidinium chloride, 10 mM dithiothreitol, 5 mM EDTA, 0.1 M Tris-HCl buffer, pH 8.0, at 37°C overnight and then alkylated with 25 mM iodoacetamide in the same buffer and then dialyzed against 4 M guanidinium chloride. After centrifugation, the supernatant was retained. This solubilised “insoluble” MUC2 complex was diluted in 4 M guanidinium chloride and 100 µl/well were coated onto microtiter plates (NUNC F96; Polysorp, Roskilde, Denmark) overnight at 4°C. The microtiter plates were washed 3 times in PBS pH 7.4 containing 0.05% Tween-20 between all ensuing steps. Unbound sites were blocked with 0.5% bovine serum albumin in 1% blocking reagent for ELISA in PBS containing 0.05% Tween-20 for 1 h. The microtiter plates were incubated with biotinylated *C. rodentium* in 1% in blocking reagent for ELISA containing 0.05% Tween pH 7.4 for 1 h at room temperature. The microtiter plates were incubated with HRP conjugated streptavidin diluted 1∶2000. Bound secondary antibody was visualized using 2,2′-azinobis(3-ethylbenzothiazoline)-6-sulphonic acid as a substrate in citric acid- Na_2_HPO_4_ buffer, pH 4.3. Absorbance at 405 nm was measured after 40 min. When the 6 M guanidinium HCl-insoluble fraction containing Muc2 was subjected to agarose gel electrophoresis and then blotted onto a membrane, the only PAS positive band present corresponded exactly to the MU2 band identified by Western blotting. However, we have not performed Western blotting for other mucins and very small amounts of other contaminating mucins cannot be excluded.

### Statistics

CFU data were log transformed. Individual data points are presented and the non-parametric Mann Whitney U test was applied to ascertain differences. All statistics were determined using Systat 12 (Systat Software, Inc., San Jose, USA).

## Results

### *C. rodentium* infection and associated pathology is greatest in the large intestine

**Figure 1:**
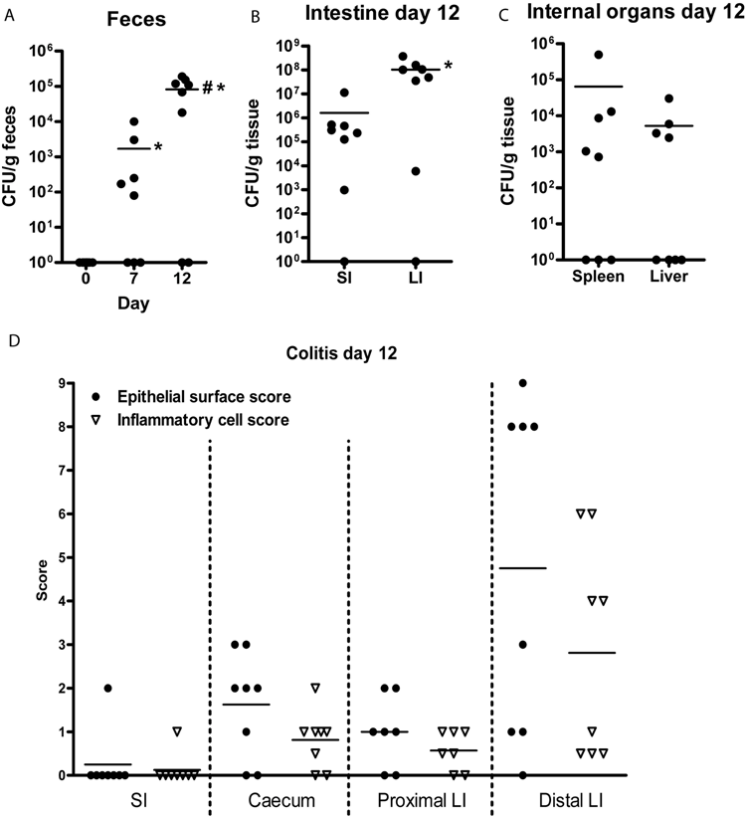
*C. rodentium* induced colitis and infection load. The number of *C. rodentium* CFU in feces increased over time (A, * p<0.05 compared to pre-inoculation, ^#^ p<0.05 compared to day 7, Mann Whitney U test, n = 8). At day 12, a higher number of CFU was detected in the large intestine (LI) than in the small intestine (SI) (B,* p<0.05), and low numbers of CFU were found in the internal organs (C). Histological colitis scores were low to moderate, with the distal large intestine being worst affected (D).

Before inoculation, *C. rodentium* was not detected in the murine feces (Figure 1A). On day 7, *C. rodentium* was detected in feces of 5/8 mice and the mean *C. rodentium* content in feces was 10^3^ CFU/g. By day 12, *C. rodentium* was detected in feces of 6/8 mice and the mean *C. rodentium* content in feces had increased to 10^5^ CFU/g feces (Figure 1A). As fecal *C. rodentium* CFU counts were maximal at 12 days and the fecal *C. rodentium* load decreased at later time points (data not shown), we sacrificed the mice on day 12. *C. rodentium* was detected in intestinal tissues of 7/8 mice with approximately 100 fold higher CFU in the colon compared to small intestine (10^8^ vs 10^6^, Figure 1B). In 6/8 animals *C. rodentium* was detected in the liver and/or spleen, demonstrating that live bacteria cross the intestinal barrier. The mice did not lose any weight, and the infection only induced mild diarrhea with no rectal bleeding at any time (data not shown).

In the small intestine, only 1/8 mice developed mild inflammation with irregular crypt architecture, 10–25% goblet cell loss, increased leukocytes in the lamina propria and slightly increased levels of polymorphonuclear cells in the lamina propria. In the caecum and proximal colon, 6/8 animals showed signs of mild colitis (Figure 1D). The distal colon was most affected by the infection with moderate crypt loss, crypt abscesses, discrete lesions, increased crypt length, >50% goblet cell loss, transmural extension of inflammatory infiltrates and high levels of polymorphonuclear cells in the lamina propria (Figure 1D). Non-infected control mice did not show any signs of colitis (data not shown).

**Figure 2:**
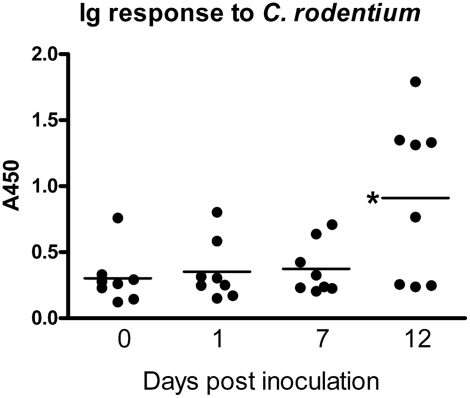
Serum Ig response to *C. rodentium*. Serum concentrations of Ig reactive with *C. rodentium* lysate determined by ELISA in 8 mice prior to and 1, 7 and 12 days following infection.

Mice infected with *C. rodentium* normally mount an IgG response that eradicates the bacteria after 3–4 weeks [43]. By day 12 most mice in this study had an increased Ig response to *C. rodentium* (Figure 2). However, at this time point the Ig response had not cleared the *C. rodentium* in any of the animals as all the animals with an increase in *C. rodentium* reactive Ig had both high intestinal CFU counts and *C. rodentium* present in their spleen and/or liver.

### Mucin expression in the healthy murine intestinal tract and following *C. rodentium* infection

Mucin expression was evaluated by Alcian blue/periodic acid Schiff (AB/PAS) staining and by immunohistochemistry for the Muc2 and Muc5AC secreted mucins, and the Muc1, Muc3/17 (hereto Muc17), Muc4 and Muc13 cell surface mucins in the small intestine, caecum, proximal-, mid- and distal large intestine of uninfected and infected mice. Muc5ac was not expressed in normal or infected intestine (stomach control sections were strongly positive).

#### Small Intestine

**Figure 3:**
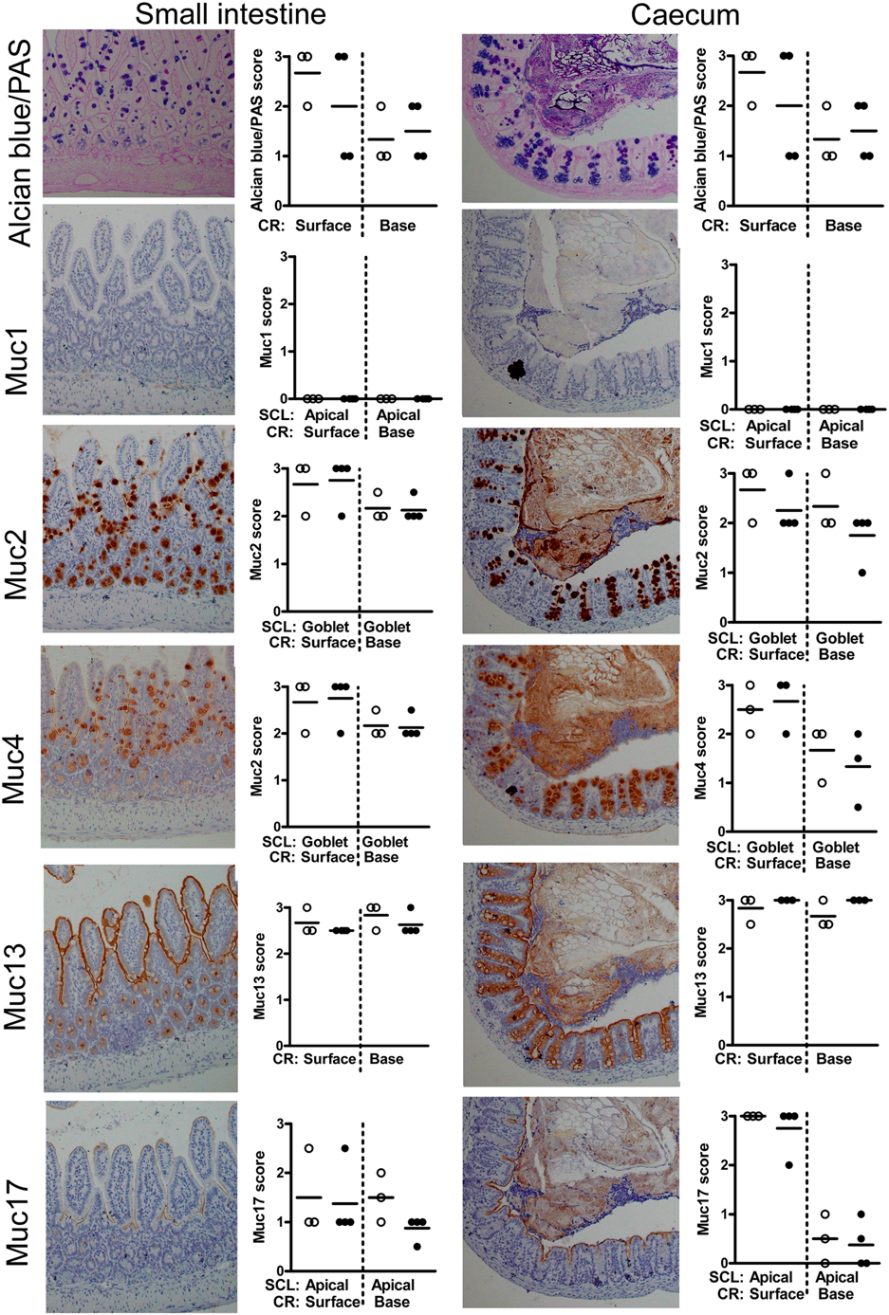
Tissue localization of mucins in small intestine and caecum. Histologically, the small intestinal villi and caecal crypts are covered by a simple columnar epithelium consisting of enterocytes with interspersed goblet cells. Using immunohistochemistry, we demonstrated that the apical surface of small intestine and caecal epithelia are lined by the cell surface mucins Muc13 and Muc17 as well as a small amount of Muc4 (brown). The goblet cells contain alcian blue positive material (blue), which mainly is Muc2 (brown), but also some Muc4. The photographs were taken using a 20× magnification. The quantification scores for any of these mucins were not significantly different between non-infected mice (○) and mice infected with *C. rodentium* for 12 days (•) in these areas of the intestine. (Mann Whitney U test, n = 7). SCL = subcellular localization, RC = region of crypt. The mucin stains are shown in serial sections on the same tissue area, whereas the mucin stain scores represent the average stain scores for the entire specimen for each individual mouse.

AB stains acidic mucins blue and the PAS stains neutral mucins pink whilst mixtures of neutral and acidic mucins appear purple. In the small intestine, the goblet cells varied from purple to blue and tended to be bluer towards the bottom of the crypts with more purple/fushia towards the top (Figure 3), indicating that the carbohydrates are more highly charged towards the bottom of the crypts. All goblet cells expressed Muc2 and most of the villous goblet cells expressed Muc4 with weaker staining in the crypt goblet cells. Enterocytes expressed the Muc4, Muc13 and Muc17 mucins, but not Muc1, with the Muc13 antibody giving the strongest stain, which decorated the surface of the entire glandular and villous epithelium. The presence of Muc4 in goblet cells and enterocytes is similar to that described for human tissue, and is consistent with previously published work showing that MUC4 can be produced both in membrane bound and soluble forms [40], [41]. No discernable changes were seen in expression of any of the mucins in the infected small intestine, despite the presence of a low density of C. rodentium.

#### Caecum

Caecal goblet cells were strongly AB positive and contained Muc2 and Muc4 (Figure 3). Muc4 was weakly expressed on the epithelial surface, whereas Muc13 was strongly expressed on the apical surface and in the cytoplasm of caecal epithelial cells and Muc17 was apically expressed on the surface cells but not in the crypts. Like the small intestine, Muc1 was not expressed in the caecum and there were no changes in mucin expression in response to infection.

#### Proximal large intestine

**Figure 4:**
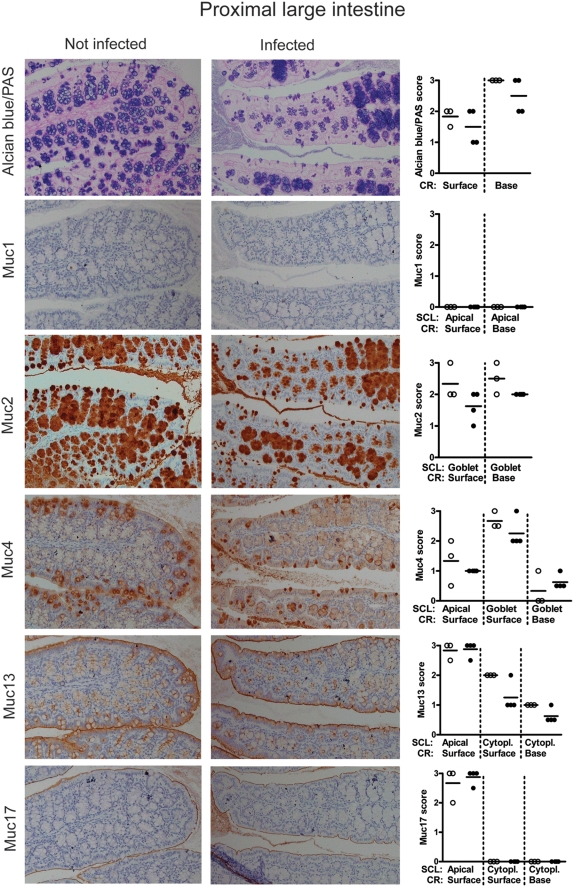
Tissue localization of mucins in the proximal large intestine. The apical surface of the epithelial cells of the crypts in the leaf-like folds of the proximal large intestine is lined by the cell surface mucins Muc4, Muc13 and Muc17 (brown). The goblet cells contain Alcian blue positive material (blue), which mainly is Muc2 (brown), although the goblet cells of the surface also contain Muc4. The photographs were taken using a 20× magnification. The quantification scores for any of these mucins were not significantly different between non-infected mice (○) and mice infected with *C. rodentium* for 12 days (•) in these areas of the intestine. (Mann Whitney U test, n = 7). SCL = subcellular localization, RC = region of crypt. The mucin stains are shown in serial sections on the same tissue area, whereas the mucin stain scores represent the average stain scores for the entire specimen for each individual mouse.

In the proximal large intestine the two distinct crypt and surface lineages of goblet cells were both strongly AB positive and contained Muc2, whereas Muc4 was present in the goblet cells on the surface, but to a lesser degree in the crypts (Figure 4). The enterocytes and their apical surfaces were both weakly positive for Muc4 and more strongly positive for Muc13, whereas Muc17 was restricted to the apical surface of the surface epithelium, and Muc1 was not expressed (Figure 4). Although no statistically significant differences in mucin expression were detected between non-infected and *C. rodentium* infected proximal colon, the Muc2/AB filled goblet cell thecae had a higher variability in size in the infected animals with groups of crypts being either hypertrophic and bulging, whilst other groups of crypts were partially depleted of mucin (Figure 4). This suggests that both Muc2 production and secretion is increased in the proximal large intestine of infected mice. Saponification prior to mild PAS staining to reveal O-acetylated mucin oligosaccharides revealed O-acetylation in a proportion of the goblet cell lineage found at the base of the crypts of the proximal and mid-colon, as previously reported [44]. Post-infection the loss of goblet cells was reflected in reduced overall mild PAS staining, however, in the remaining cells of the basal goblet cell lineage there was an increase in mild PAS staining without prior saponification, indicating a reduction of O-acetylation in those cells following infection (Figure S1).

#### Mid and distal large intestine

**Figure 5:**
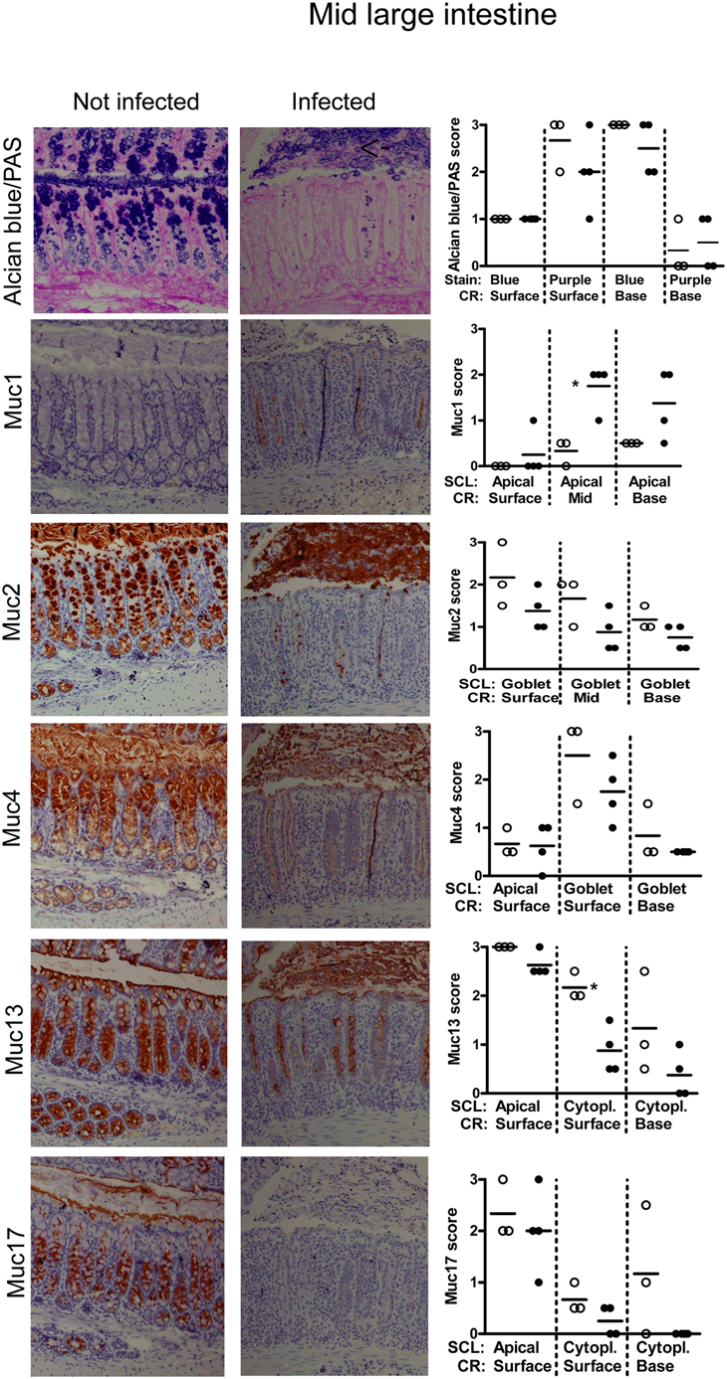
Tissue localization of mucins in the mid large intestine. The apical surface of the mid large intestinal epithelium and crypts are lined by the cell surface mucins Muc13 and Muc17 as well as a small amount of Muc4 (brown). The goblet cells contain Alcian blue positive material (blue = highly charged carbohydrate structures, purple = medium charged carbohydrate structures), which mainly is Muc2 (brown) and Muc4. The photographs were taken using a 20× magnification. The quantification scores for Muc1, and Muc13 were significantly different between non-infected mice (○) and mice infected with *C. rodentium* for 12 days (•) (* p<0.05, Mann Whitney U test, n = 7). SCL = subcellular localization, RC = region of crypt. The mucin stains are shown in serial sections on the same tissue area, whereas the mucin stain scores represent the average stain scores for the entire specimen for each individual mouse.

**Figure 6:**
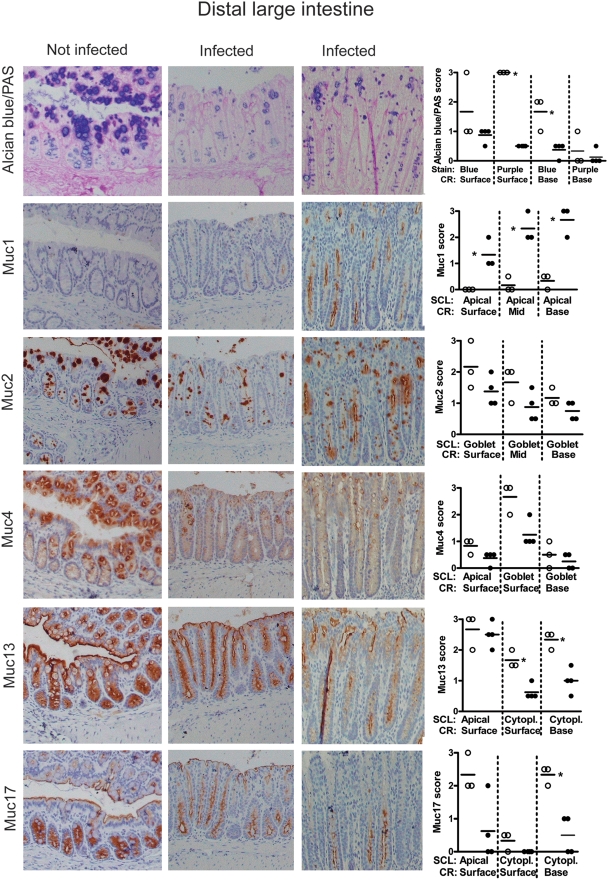
Tissue localization of mucins in the distal large intestine. The apical surface of the distal large intestinal epithelium and crypts are lined by the cell surface mucins Muc13 and Muc17 as well as a small amount of Muc4 (brown). The goblet cells contain Alcian blue positive material (blue = highly charged carbohydrate structures, purple = medium charged carbohydrate structures), which mainly is Muc2 (brown), although the goblet cells of the surface also contain Muc4. The photographs were taken using a 20× magnification. The quantification scores for Alcian blue, Muc1, Muc13 and Muc17 were significantly different between non-infected mice (○) and mice infected with *C. rodentium* for 12 days (•) (* p<0.05, Mann Whitney U test, n = 7). SCL = subcellular localization, RC = region of crypt. The mucin stains are shown in serial sections on the same tissue area, whereas the mucin stain scores represent the average stain scores for the entire specimen for each individual mouse.

In the mid and distal large intestine, the upper half of the crypts contain the majority of the goblet cells, whereas the lower half of the crypts contain cells of secretory type but with a more vacuolated morphology [45]. AB/PAS staining of the goblet cells revealed medium charged carbohydrates from the middle of the crypt to the top, whereas the bottom of the crypts had a small amount of highly charged carbohydrate chains (Figures 5 and 6). As for the other regions of the intestine these goblet cells expressed Muc2 and to a lesser extent Muc4. In the mid- and distal colon, the apical surface of the lower half of the crypt was weakly positive for Muc1 (Figures 5–6 and Figure S2, intensity score 0–0.5). Muc13 and Muc17 were highly expressed in the cytoplasm of the cells with secretory vacuolated morphology in lower half of the crypt and also expressed on the apical surface of cells in the crypts and the surface epithelium (Figure 6). Muc17 was also present cytoplasmically in the bottom half of the crypt (Figures 5–6). Thus, the tissue localization of Muc17 is similar to that of Muc13 in the mid and distal large intestine, whereas differences occur in the more proximal parts of the intestine.

*C. rodentium* caused focal damage and elongation of crypts in the mid and distal colon, with patches of almost normal epithelium interspersed with the more inflamed elongated crypts. In infected animals, the distal colonic crypts were depleted of AB, Muc2 and Muc4 positive material (p<0.05 for AB/PAS, Figure 6). In some areas the goblet cell thecae were depleted from the base of crypts but some goblet cell thecae remained at the top of crypts, and there was a massive amount of secreted mucus and shed goblet cells (see arrow in Figure 5) in the lumen. The mid large intestine also tended to be depleted of stored Alcian blue positive material at the bottom of the crypt and partially of the purple goblets from mid length to top (Figure 6), although the variability between animals was larger in this location. The more elongated crypts tended to be more depleted of mucins linking goblet cell depletion with localized infection and/or inflammation.

In the *C. rodentium* infected animals the apical Muc1 expression in the lower half of the crypt was increased in the mid and distal colon, and de novo expression occurred apically on the surface epithelium in 3/4 animals (p<0.05, Figures 5–6 and Supplementary Figure 1). Although the apical Muc13 expression was similar to that of non-infected animals, the cytoplasmic expression was significantly decreased (Figures 5–6), consistent with increased apical turn-over of this cell surface mucin. Similarly, Muc17 levels in the cytoplasm of the crypt base also decreased in the mid and distal large intestine with infection and the apical expression in the surface epithelium also decreased (Figures 5–6).

### *C. rodentium* binding to Muc2

**Figure 7:**
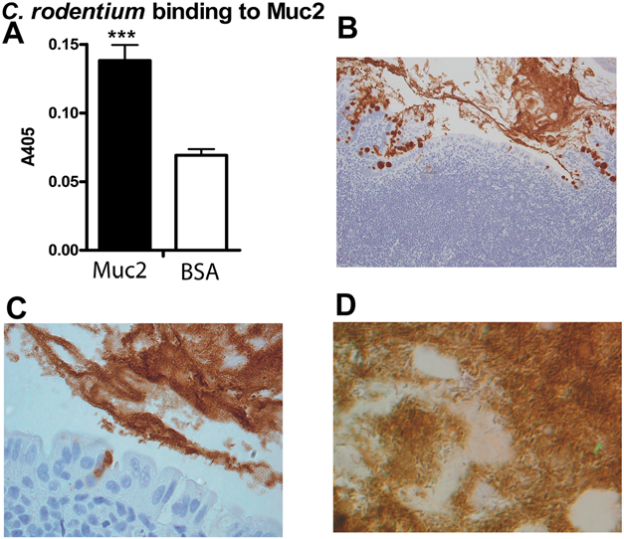
*C. rodentium* binding to Muc2. ELISA plates were coated with the “insoluble” Muc2 complex or with BSA and incubated with biotinylated *C. rodentium*. Statistics: Mean±SD, *** p<0.001 (t-test, n = 6) (A). Large amounts of bacteria can be found entangled in the secreted Muc2 in *C. rodentium* infected animals (B–D). By brightfield observation using a 20× lens (B) the Muc2 is visualized as a brown stain, and the presence of large amounts of bacteria within the secreted Muc2 can be seen by reflected light Nomarski differential interference observation (C; 40× lens and D; 100× lens).

In the healthy intestine, Muc2 is the main secreted mucin composing the mucus layer, which is continuously washing the intestinal surface to clear trapped material. Using a microtiter based assay, we demonstrated that murine Muc2 can bind to *C. rodentium* (Figure 7A). Although it cannot be excluded that the insoluble Muc2 complex used in this study could contain small amounts of contamination with other reduction sensitive polymeric complexes (for example other mucins), large amounts of bacteria were found in the secreted Muc2 in *C. rodentium* infected animals (Figure 7B–D), consistent with Muc2 binding to *C. rodentium*.

### *Salmonella* St Paul, *Campylobacter jejuni* and *Clostridium difficile* induce MUC1 in human distal colon during infection

**Figure 8:**
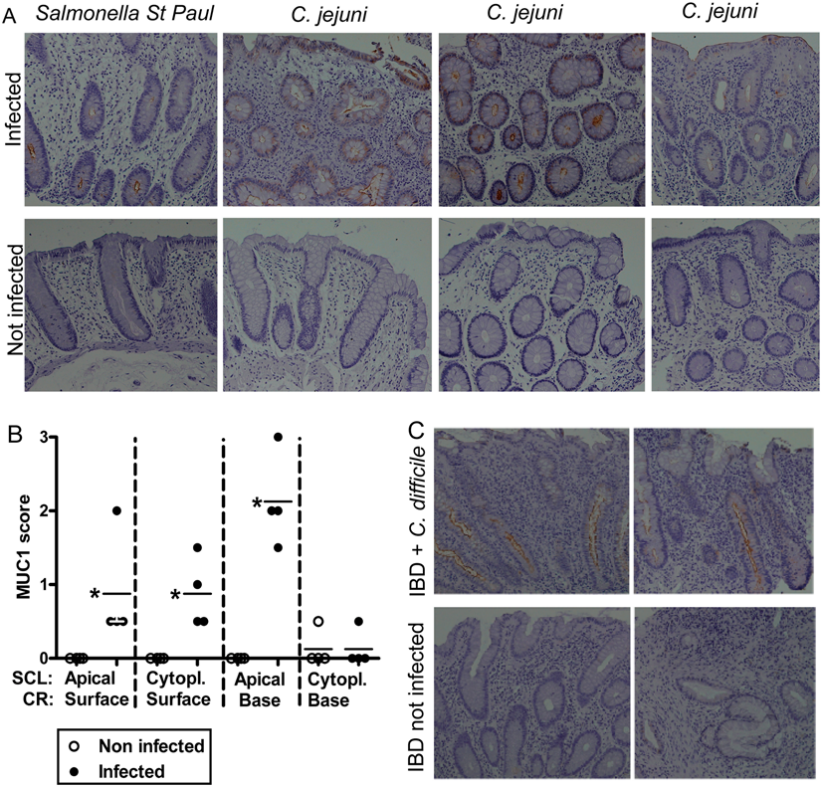
Induction of MUC1 in human intestinal infection. MUC1 expression in distal colon in humans with *S.* St Paul (upper left panel) and *C. jejuni* (3 upper right panels) gastroenteritis (A). The lower panels show lack of MUC1 in non-infected age, sex and colonic site matched controls. The MUC1 quantification scores (B) were higher in colonic biopsies from infected (•) than non-infected (○) patients (* p<0.05, Mann Whitney U test, n = 8). Colonic biopsies from two IBD patients with acute *C. difficile* infection demonstrated that MUC1 is higher during infection even in the background of chronic inflammation. The photographs were taken using 20× magnification.

Distal colonic biopsies from four patients infected with *S.* St Paul or *C. jejuni* were positive for MUC1, whereas the age, sex and site matched control samples had no or very little detectable MUC1 (p<0.05, Figure 8A and B). Similarly, the MUC1 expression in distal colonic biopsies from two IBD patients with acute *C. difficile* had higher levels of MUC1 compared to biopsies from the same site in the absence of *C. difficile* infection, although low levels of MUC1 were still present in the IBD control biopsies (Figure 8C). No MUC1 was demonstrated in one IBD patient infected with *Salmonella enteritidis*. Thus, MUC1 is induced in response to infection by a range of different intestinal pathogens in humans, in a similar manner to the induction we demonstrated experimentally in *C. rodentium* infection in mice.

## Discussion

This work demonstrates that six mucins have distinctly different expression patterns throughout the murine intestinal tract, indicating that they are differentially regulated and are likely to make different functional contributions to the mucosal barrier. The cellular content of most mucins changes in response to infection, with Muc1 being the only mucin to be induced whereas all other mucins were depleted, albeit to differing degrees. As mucins are strategically positioned between the vulnerable mucosa and the bacterial contents of the intestinal lumen [46], the changes in mucin concentrations and the consequent changes in mucin binding to bacteria are likely to affect both host-commensal and host-pathogen interactions.

Muc1/MUC1 appears to play a significant role in host defense in acute infection. In the murine infection model of *C. jejuni*, Muc1 is upregulated acutely in the intestine in response to infection [6]. We now show that *C. rodentium* infection also induces Muc1 in mouse colon, and similarly that *S.* St Paul, *C. jejuni* and *C. difficile* induced MUC1 in human colon during infection. Thus, Muc1/MUC1 is induced in response to infection by a range of different pathogens in both mice and humans. As Muc1^−/−^ mice have a higher rate of systemic *C. jejuni* infection [6], Muc1/MUC1 induction appears to be an effective host defense used by both human and mouse innate immunity. MUC1 expression in biopsies from inflammatory bowel disease patients with chronic inflammation was also higher during acute infection compared to the inflammatory bowel disease control biopsies. This indicates that MUC1 is upregulated in response to acute infection and that chronic inflammation by itself is not sufficient to substantially upregulate MUC1.

Previous studies have shown that mucins bind to several pathogens [6], [22], [23] and we show here that mucins bind to *C. rodentium*. Increased secretion of mucin in response to infection has been demonstrated using *in vitro* airway epithelial cells co-cultured with *Burkholderia cenocepacia* and *Listeria monocytogenes* inoculated rat ileal loops [47], [48]. The depletion in the amount of Alcian blue positive mucus material as well as Muc2, Muc4, Muc13 and Muc17 we detected here is also likely to occur as a consequence of increased secretion and therefore be part of the host defence. In a recently published study, Muc2 depletion in *C. rodentium* infected mice was shown to be dependent on T- and B-cells [49]. However, for such a host defence to be effective the mucus must be replenished. In our infected specimens we saw large amounts of shed whole cells, including goblet cells (Figure 5). The factors causing premature death of the goblet cells with infection are unknown, although it appears that *C. rodentium* directly interact with and infect murine goblet cells during infection [49] and may therefore result in goblet cell death. Shedding of these cells would delay replenishment of the mucus layer providing an advantage to the mucosal pathogen. In addition, several pathogens have been shown to specifically inhibit mucin synthesis [50], [51]. A further possibility is that the goblet cells experience endoplasmic reticulum stress due to the enormous amount of protein synthesis that they effect during infection and that the consequent unfolded protein response shuts down mucin production and if more prolonged or severe causes apoptosis [39], [52], [53]. It seems likely that the host attempts to remove pathogens by increasing secretion of mucins to trap and remove pathogens from the epithelial surface, whereas the pathogen tries to eliminate this host defence by inhibiting the mucin synthesis and causing shedding of the mucus producing epithelial cells. Interestingly, goblet cell depletion during infection does not appear to occur in the respiratory tract where goblet cell hyperplasia and mucus hypersecretion can continue for extended periods [54]. Furthemore, changes in mucin secretion is likely to affect the resident microflora due to the large change in glycosylated compounds available for bacterial degradation [55], [56].

In the small intestine and proximal large intestine, *C. rodentium* was present, albeit in lower amounts than in the distal large intestine, and yet there were few signs of mucosal damage, inflammation or changes in mucins. Furthermore the highest concentrations of bacteria were detected in the colons of animals with the highest amount of colonic damage, mucin depletion, and systemic infection. Even within animals, damage and inflammation in the distal colon was often patchy suggesting that focal bacterial penetration of the barrier leads to localised mucin release and inflammatory responses. Why these bacteria penetrate the barrier in the distal colon remains to be elucidated but could be due to an increased bacterial pathogen load in the distal colonic niche, or an ability to degrade the mucin glycoforms in this region, and/or involve induction of pathogenicity-related genes in the bacteria due to local environmental cues. We have demonstrated that MUC2 is one such cue for *C. jejuni* which switches on pathogenicity genes in response to mucin exposure [57]. An IgG response has been shown to be integral to clearance of *C. rodentium* infection [58] and it appears pertinent for our understanding of the onset of humoral immunity that the *C. rodentium* specific Ig production at this stage of infection was restricted to those mice in which the bacteria had penetrated the mucosal barrier.

*C. rodentium* infection causes colonic inflammation, mucosal hyperplasia, epithelial dysfunction in association with increased permeability to luminal bacteria and a vigorous Th1 inflammatory response [59]. Thus *C. rodentium* infection elicits mucosal inflammation with similarities to inflammatory bowel disease, and has therefore been used to investigate the relationship between inflammation and anti-bacterial immunity in the gut [4]. MUC5AC, which is a product of normal gastric mucosa, is absent from normal colon but frequently present in colorectal adenomas and colon cancers [60], [61], and expressed in patients with ulcerative colitis [61]. MUC5AC was not detected in the infected mice in our study, suggesting that induction of MUC5AC may arise from more long term effects of chronic inflammation. However, similar to our findings in murine *C. rodentium* infection, a reduction in MUC2 expression occurs in UC adjacent to ulceration and in active colitis [62], [63], and MUC1 expression was upregulated in severe UC at the site of rupture of crypt abscesses [63]. Our non-infected human biopsies from IBD patients only had a very low amount of MUC1, but they did not contain any crypt abscesses, which may induce MUC1 in a similar manner to acute infection.

In conclusion, in response to intestinal infection mucin secretion is increased and most mucins are depleted from the epithelium whilst MUC1 is upregulated. As mucins bind pathogens, increasing the secretion of mucins is likely to aid the host by trapping and removing pathogens from the epithelial surface. Pathogens may subvert this host defence by inhibiting mucin synthesis and secretion or causing premature shedding of the mucus producing epithelial cells. Further exploration of the dynamic host mucin – bacterial interactions that occur in the mucin covered mucosal interface will increase our understanding of human susceptibility to infection.

## Supporting Information

Table S1 Histological scoring of murine colitis.
(0.03 MB DOC)

Figure S1 Mild PAS staining with and without prior saponification on a non-infected and C. rodentium infected mouse.
(7.16 MB TIF)

Figure S2 Expression of Muc1 in the distal large intestine is upregulated in response to infection with C. rodentium. Representative examples of Muc1 expression determined by immunohistochemistry in the distal colon of mice infected with C. rodentium for 12 days and non-infected mice. The photographs were taken using 20× magnification.
(26.10 MB TIF)
