# Supplementary material for: Mucin Dynamics in Intestinal Bacterial Infection
Source: PLoS One. 2008 Dec 17;3(12):e3952. doi: 10.1371/journal.pone.0003952 (PMC2601037; doi:10.1371/journal.pone.0003952)
Supplement: Table S1 — Histological scoring of murine colitis. (0.03 MB DOC) [file pone.0003952.s001.doc]

**Supplementary Table**

Table S1. Histological scoring of murine colitis.

A. Large Intestine Inflammation and Damage Scores

Crypt architecture

0 = Normal

1 = irregular

2 = Moderate crypt loss (10-50%)

3 = Severe crypt loss (50-90%)

4 = Small-medium size ulcers (10 crypt widths)

5 = Large ulcers (more than 10 crypt widths)

Crypt Length

Caecum – 0 = < 150 um, 1 = 150-200, 2 = 200-250, 3 = 250-300, 4 >300

PC – 0 = < 150 uM, 1 = 150-200 um, 2 = 200-250, 3 = 250-300, 4 = >300

DC – 0 = < 250, 1 = 250-300, 2 = 300-350, 3= 350-400, 4 = >400

Crypt abscesses

0 = no crypt abscesses

1 =1-5

2 = 6-10

3 = >10

Tissue Damage

0 = No damage

1 = Discrete lesion

2 = Mucosal erosion

3 = Extensive mucosal damage

Goblet Cell Loss

0 = normal

<10% loss

1 = 10-25%

2 = 25-50%

3 = >50%

Inflammatory cell infiltration

0 = Occasional infiltration

1 = Increasing leukocytes in lamina propria

2 = Confluence of leukocytes extending to sub-mucosa

3 = Transmural extension of inflammatory infiltrate

Lamina propria neutrophils (PMN)

0 = 0-5 PMNs

1 = 6-10 PMNs

2 = 11-20 PMNs

3 = > 20 PMNs

PMNs scored from 10 HPF areas

B. Small Intestinal Damage Inflammation Scores

Crypt and villous architecture

0 = Normal

1 = irregular

2 = Shortening of villi

3 = Some villi completely lost, crypt length increased

Inflammatory cell infiltration

0 = Occasional infiltration

1 = Increasing leukocytes in lamina propria

2 = Prominent leukocytes in villi and between glands

3 = Transmural extension of inflammatory infiltrate
