# Supplementary figures and images for: Mucin Dynamics in Intestinal Bacterial Infection
Source: PLoS One. 2008 Dec 17;3(12):e3952. doi: 10.1371/journal.pone.0003952 (PMC2601037; doi:10.1371/journal.pone.0003952)

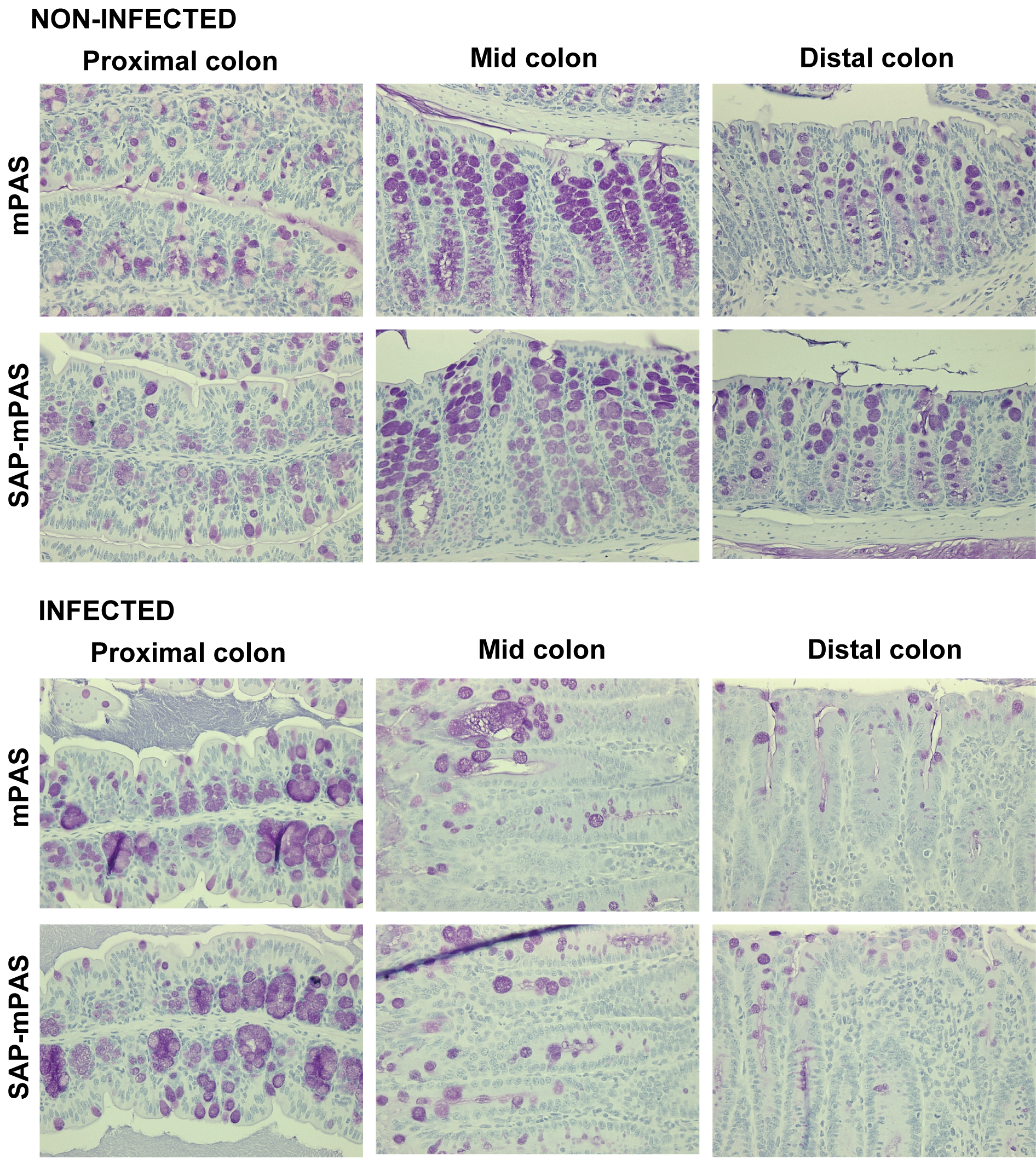

Supplement: Figure S1 — Mild PAS staining with and without prior saponification on a non-infected and C. rodentium infected mouse. (7.16 MB TIF) [file pone.0003952.s002.tif]

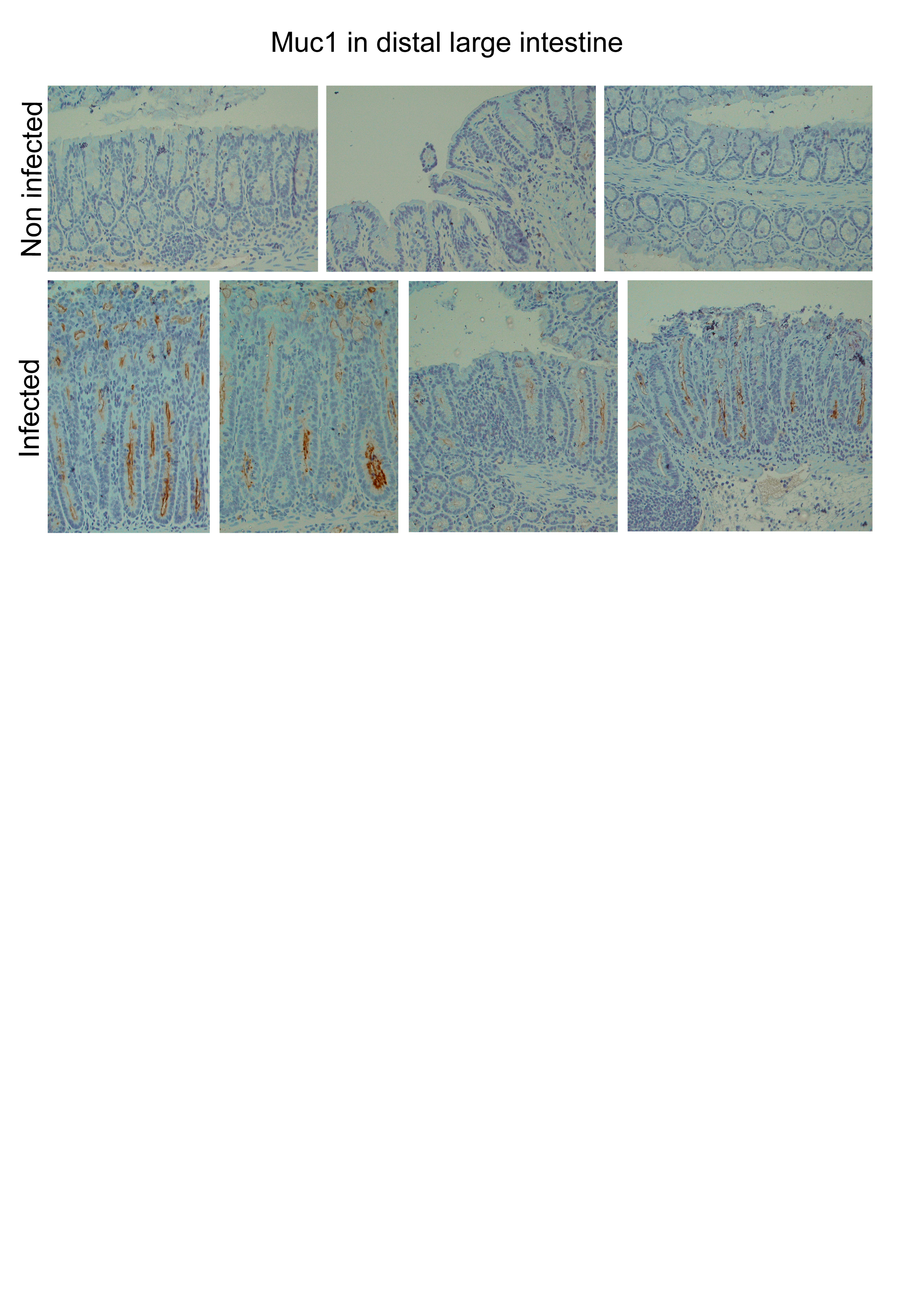

Supplement: Figure S2 — Expression of Muc1 in the distal large intestine is upregulated in response to infection with C. rodentium. Representative examples of Muc1 expression determined by immunohistochemistry in the distal colon of mice infected with C. rodentium for 12 days and non-infected mice. The photographs were taken using 20× magnification. (26.10 MB TIF) [file pone.0003952.s003.tif]
